# Interventions among Pregnant Women in the Field of Music Therapy: A Systematic Review

**DOI:** 10.1055/s-0041-1731924

**Published:** 2021-06-28

**Authors:** Bruna Mayumi Omori Shimada, Magda da Silva Oliveira Menezes dos Santos, Mayara Alvares Cabral, Vanessa Oliveira Silva, Gislaine Cristina Vagetti

**Affiliations:** 1 Universidade Estadual do Paraná (UNESPAR), Curitiba, Paraná, PR, Brazil

**Keywords:** pregnancy, music therapy, music, women's health, gravidez, musicoterapia, música, saúde da mulher

## Abstract

**Objective** To investigate in the literature the studies on the benefits of music therapy interventions among pregnant women in the prenatal, delivery and postpartum periods.

**Data Sources** The search for articles was carried out in the following electronic databases: VHL, LILACS, SciELO, Portal CAPES, PsycINFO, ERIC, PubMed/Medline, and journals specialized in this field: *Revista Brasileira de Musicoterapia* (“Brazilian Journal of Music Therapy”) and *Voices* .

**Study Selection** Descriptors in Portuguese ( *musicoterapia* , *gravidez* , *gestantes* , *revisão* ), English ( *music therapy* , *pregnancy* , *pregnant women* , *review* ) and Spanish ( *musicoterapi* a, *embarazo* , *mujeres embarazadas* , *revisión)* were used. The search was delimited between January 2009 and June 2019. The process of selection and evaluation of the articles was performed through peer review.

**Data Collection** The following data were extracted: article title, year of publication, journal, author(s), database, country and date of collection, purpose of the study, sample size, type of care, intervention, instruments used, results, and conclusion. The data were organized in chronological order based on the year of publication of the study.

**Summary of the Data** In total, 146 articles were identified, and only 23 studies were included in this systematic review. The articles found indicate among their results relaxation, decreased levels of anxiety, psychosocial stress and depression, decreased pain, increase in the maternal bond, improvement in the quality of sleep, control of the fetal heart rate and maternal blood pressure, and decreased intake of drugs in the postoperative period.

**Conclusion** Music therapy during the prenatal, delivery and postpartum periods can provide benefits to pregnant women and newborns, thus justifying its importance in this field.

## Resumo

**Objetivo** Investigar na literatura os estudos sobre os benefícios das intervenções musicoterapêuticas em gestantes no pré-natal, parto e pós-parto.

**Fontes dos dados** A busca dos artigos foi realizada nas seguintes bases de dados eletrônicas: BVS, LILACS, SciELO, Portal CAPES, PsycINFO, ERIC, PubMed/Medline e revistas especializadas da área: *Revista Brasileira de Musicoterapia* e *Voices* .

**Seleção dos estudos** Utilizaram-se descritores em português ( *musicoterapia* , *gravidez* , *gestantes* , *revisão* ), em inglês ( *music therapy* , *pregnancy* , *pregnant women* , *review* ) e em espanhol ( *musicoterapia* , *embarazo* , *mujeres embarazadas* , *revisión* ). A busca foi delimitada de janeiro de 2009 até junho de 2019. Os processos de seleção e avaliação dos artigos foram realizados por revisão por pares.

**Coleta de dados** Os seguintes dados foram extraídos: título do artigo, ano da publicação, revista, autor(es), base de dados, país e data da coleta, objetivo do estudo, tamanho da amostra, tipo de atendimento, intervenção, instrumentos utilizados, resultados, e conclusão. Os dados foram organizados em ordem cronológica a partir do ano de publicação do estudo.

**Síntese dos dados** Foram identificados 146 artigos e incluídos apenas 23 estudos na revisão sistemática. Os artigos encontrados indicam em seus resultados relaxamento, diminuição dos níveis de ansiedade, de estresse psicossocial e de depressão, diminuição da dor, aumento do vínculo materno, melhora da qualidade do sono, controle da frequência cardíaca fetal e da pressão arterial materna, e diminuição da ingestão de fármacos no pós-operatório.

**Conclusões** A musicoterapia durante o pré-natal, parto e pós-parto pode trazer benefícios para a gestante e para o neonato, o que justifica sua importância nessa área.

## Introduction

Pregnancy is a period characterized by physical, hormonal and emotional changes. 1 The birth marks a new phase in the life of the woman, the puerperium, which ends when the woman's body returns to the stage previous to pregnancy. 2

Pregnant women are especially affected by stress during pregnancy, childbirth and the postpartum period. Several art forms have been studied in order to evaluate their relaxing potential and their effects on the physiology of individuals. Music has been a constant target of research regarding its effects on the most diverse groups of patients. The existing data point to its importance in improving the concentration, attention and physical endurance of the patients. 3 And it has been shown to be beneficial in the emotional, intellectual, psychological, physiological and social fields, 4 in addition to having specific beneficial effects regarding depression and normal postpartum pain, anxiety and greater satisfaction in the postpartum period. 5

In pregnant women, these effects can be explained by a series of physiological mechanisms that are activated at the moment of listening to music, which remain activated for a prolonged period. As the main neurotransmitters related to music therapy, natural serotonin – which creates a state of relaxation – and acetylcholine have their potential boosted, with an effect of reducing the heart rate and blood pressure, and increasing blood flow to noble organs. 6 Listening to music also causes the glucocorticoids such as cortisol, which are strongly related to the state of stress, to have a reduced release, with a consequent benefit regarding fetal development, since they are able to cross the placental barrier and directly interfere in fetal physiology. 7

A form of treatment that aims at the physical, mental and psychological integration of the patient, music therapy is also one of the methods used as a support in pregnancy. Some studies in the literature have shown that musical interventions have an insignificant effect on the reduction in stress during pregnancy 8 and in the decrease in pain during childbirth. 9 However, there a significant improvement in the levels of anxiety during pregnancy and labor has been observed. 8 9 A systematic review by Van Willenswaard et al. 8 points out that no study on music therapy was found during their detailed search, diverging from other systematic reviews that examined interventions made by a music therapist. In view of the divergent results in the literature, the importance of the present study is evident. Therefore, the aim of the current study was to investigate in the literature about the benefits of music therapy interventions among pregnant women in the prenatal, delivery and postpartum periods.

## Methods

### Type of study

The present is a systematic literature review performed according to the Preferred Reporting Items for Systematic Reviews and Meta-Analyses (PRISMA) statement. 10 The methodology of a systematic review has a high performance in identifying scientific evidence. According to the Oxford Center for Evidence-Based Medicine (OCEBM), 11 the typology of the systematic review is classified as level 1 out of 5 possible levels in the representation of evidence, as it makes it possible to establish a panorama on the studied topic. We used the The State of the Art through Systematic Review (StArt) software, developed by the Software Engineering Research Laboratory (Laboratório de Pesquisa em Engenharia de Software, LAPES, in Portuguese) of Universidade Federal de São Carlos, Brazil, which supports the researcher in the systematic review.

### Search strategy

The research question was formulated using the “PICO” framework, which means population (participants), intervention (or exposure, for observational studies), comparison, and “outcome”, with some researchers preferring to add and “S” (for study design), therefore naming it PICOS. 12

The search was carried out in the following electronic databases: Biblioteca Virtual de Saúde (BVS), Literatura Latino-Americana e do Caribe em Ciências da Saúde (LILACS), Scientific Eletronic Library Online (SciELO), Portal CAPES, PsycINFO, Education Resources Information Center (ERIC), PubMed/Medline, and in journals specialized in the field: *Revista Brasileira de Musicoterapia* (“Brazilian Journal of Music Therapy”) and *Voices* .

It was delimited from January 2009 to June 2019, considering articles published in Portuguese, English and Spanish. We used descriptors in Health Sciences (DeCS), Medical Subject Headings (MeSH) and Thesaurus in Portuguese ( *musicoterapia* , *gravidez* , *gestantes* , *revisão* ), in English ( *music therapy* , *pregnancy* , *pregnant women* , *review* ) and in Spanish ( *musicoterapia* , *embarazo* , *mujeres embarazadas* , *revisión* ). Descriptors were combined using the Boolean operators “AND” and “OR”.

The articles were selected and evaluated by peer review and organized in phases: in the first phase, an initial analysis of the titles of the manuscripts was carried out; in the second phase, an evaluation of the abstracts was performed. In the third phase, all selected articles were obtained in full, and were subsequently examined according to the established inclusion and exclusion criteria.

### Inclusion and exclusion criteria

The inclusion criteria were: original articles published in journals; and studies published from January 2009 until June 2019. The exclusion criteria were: theses, dissertations, monographs and studies that did not reach a minimum score of 18 points in the Downs and Black 13 checklist. If differences occurred during the review of the articles, new discussions were held until both reviewers agreed with the review.

### Data extraction

The following data were extracted from the articles included: title, year of publication, journal, author(s), database, country and date of collection, objective of the study, sample size, type of care, intervention, instruments used, results, and conclusion. The articles were organized in chronological order based on the year of publication.

## Results

**Fig. 1.**
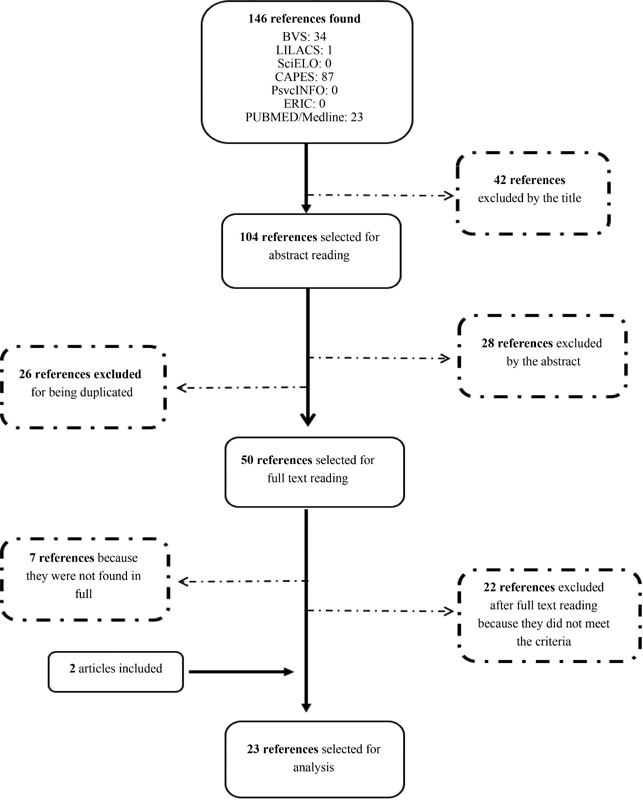
Flowchart of the process of search and selection of studies.

Figure 1 presents a flowchart of the search and selection process. In the analysis of the titles, 104 studies were selected and had their abstracts read; 50 studies were considered relevant, and their full texts were read. Out of these studies, 22 were excluded because they did not meet the eligibility criteria, and 7 references were excluded because they were not found in full. During the search, we included two articles found in the references of other articles. The electronic search generated 23 studies, with 8 articles on childbirth and postpartum, and 15 studies related to prenatal care ( Figure 1 ).

## Discussion

Regarding the studies selected ( Table 1 ), 6 were from Turkey, and they reported decreased anxiety, FCF, PA, and postoperative pain; 5 14 15 16 17 18 3 were from Brazil, with results regarding the reduction of pain; 1 19 20 3 were from Taiwan, and they reported decreased stress and anxiety, improved quality of sleep, and decreased pain in the initial phase of labor 21 22 23 ; 3 were from Spain, with results describing decreased anxiety, PAS, PAD and HR and fetal reactivity 4 7 24 ; 1 was from Ireland, and the researchers achieved relaxation and increased bonding; 25 1 was from the United Kingdom, and it reported decreased anxiety and prenatal depression; 26 1 ws from China, and the researchers found decreased anxiety and physiological responses. 27 1 was from the United States, reporting reduced suffering before delivery 28 ; 1 was from India, and the researchers obtained fetal stimulation; 29 1 was from South Korea, reporting decreased anxiety and FCF 30 ; 1 was from Israel, reporting an increase in positive emotions and a decrease in negative emotions; 31 and 1 was from Iran, reporting lower pain scores. 32

**Table 1:** Summary of the articles selected for the systematic review

**Title, author, year**	**Objective**	**Country,** **year of collection, and sample size**	**Type of care and intervention**	**Instruments used**	**Results**	**Conclusion**
**PRENATAL PERIOD**						
Music therapy to relieve anxiety in pregnant women on bedrest: a randomized, controlled trial; Yang et al. (2009) 27	To explore the effect of music therapy on anxiety relief for pregnant women on bedrest	China; 2007; 120 pregnant women	Group care Duration: 3 consecutive days. Description: the pregnant women in the experimental group received music therapy for 30 minutes on 3 consecutive days. Pregnant women who received routine care had a 30-minute rest on 3 consecutive days. The variables included anxiety and physiological responses	State-Trait Anxiety Inventory	Anxiety levels decreased and physiological responses improved significantly in the intervention group, which received music therapy during bedrest	Music carefully selected according to the preference of the pregnant woman is an inexpensive and effective method to reduce anxiety in women with high-risk pregnancies and who are on bedrest
Alleviating distress during antepartum hospitalization: a randomized controlled trial of music and recreation therapy; Bauer et al. (2010) 28	To examine the effectiveness of a single music session or intervention with recreation therapy to reduce antepartum-related suffering among women with high-risk pregnancies who experience prolonged antepartum hospitalizations	USA/2009 61 pregnant women	Individual care. Duration: before and after, within 48 to 72 hours after delivery. Description: randomized, single-blinded study; participants received 1 hour of music or recreational therapy, or were placed in an attention control group. Suffering related to antepartum was measured by the Emotional Impact Inventory of Antepartum Rest, which was administered before and after the intervention and in a follow-up period of between 48 and 72 hours	Antepartum Bedrest Emotional Impact Inventory	Significant associations were found between the provision of music and recreational therapy and the reduction of suffering related to the antepartum in women hospitalized with high-risk pregnancies. These statistically significant reductions in suffering persisted over a period of 48 to 72 hours	Music interventions in a single recreational therapy session effectively alleviate antepartum-related suffering among high-risk women who undergo hospitalization before delivery, and should be considered as a complement to any comprehensive antepartum program
Novel method of feto-maternal monitoring using music therapy - a non-stress test; Kumar et al. (2011) 29	To monitor fetal movements with and without music	India; 2010; 9 pregnant women	Individual care. Duration: not mentioned. Description: the music was set to be heard on a walkman and, the headphones were placed around the pregnant woman's abdomen. The volume of the music was kept at a moderate level of no more than 70 decibels. Fetal movements were measured by pressure sensors. The voltages obtained with and without music were amplified by the AD620 and fed to the NI 6015 for the purpose of monitoring and storage on the PC with a sampling time of 200 ms using the Labview environment	Not mentioned	With music, it increased from 146 bpm to 169 bpm. The test is reactive if there is a minimum 10-15 bpm increase in normal heart rate during fetal movements, otherwise the test is not reactive. This state was also verified by ultrasound. This test is reactive and good for the health of the fetus	Music can serve as a means of communication with the fetus through sounds and voices. Caressing the fetus through the belly, producing soft and melodic sounds, using lights and vibrations that are pleasant for the baby: these stimuli are in an organized and pleasant pattern
Effect of maternal anxiety and music on fetal movements and fetal heart rate patterns; Kafali et al. (2011) 14	To investigate the effect of the non-stress test and music on maternal anxiety and the effect of maternal anxiety and music on fetal heart rate changes.	Turkey; 2009; 201 pregnant women. Group with music = 96; group without music = 105	Group and individual care Duration: not mentioned. Description: pregnant women who came for routine prenatal care were randomized to receive music (n = 96) or no music (n = 105) during the non-stress test. Before and after the test, these women were asked to complete the Spielberg State-Trait Anxiety Inventory in two interviews; the primary outcome was considered maternal state anxiety scores before and after the non-stress test. The secondary outcome was the baseline fetal heart rate, the number of fetal movements, major accelerations, dubious non-stress test, variable decelerations, and the minimum procedure time.	State-Trait Anxiety Inventory	Before the non-stress test, the average state-trait anxiety scores of the music and control groups were of 38.1 ± 8.8 and 38.08 ± 8.2 respectively. On the other hand, after the non-stress test, the average state-trait anxiety scores of the music and control groups were of 35.5 ± 8.2 and 40.2 ± 9.2 respectively. While in the control group the non-stress trest brought about a statistically significant increase in the state-trait anxiety scores, listening to music during the non-stress test resulted in a decrease in state-trait of anxiety scores in the study group; however it was not statistically significant. The baseline fetal heart rate of the music group was significantly higher than that of the control group	The non-stress test has anxiogenic effects on mothers and listening to music, and a positive impact on maternal and fetal parameters, but it is an open question whether maternal anxiety during pregnancy can affect fetal accelerations to the point of influencing clinical judgment
The Limerick Lullaby project: an intervention to relieve prenatal stress; Carolan et al. (2012) 25	To explore the impact of lullaby singing during pregnancy	Ireland; 2009; 6 pregnant women	Group care. Duration: 4 sessions. Description: the pregnant women were recruited in childbirth classes at a maternity hospital. Six pregnant women participated and learned to sing three lullabies in four group sessions with musicians. In-depth qualitative views were taken approximately three months later to capture the experiences of the women.	Questionnaire with open questions	They suggest that learning to sing lullabies during pregnancy has benefited women in terms of relaxation, feeling closer to the fetus, connecting with other pregnant women, and providing an additional tool for communication at the beginning of the newborn period. Some women described a deep feeling of love and connection with the fetus while singing the lullabies	The main advantage of this intervention is that it is non-pharmacological and easy to implement. At the same time, it appears to be a pleasurable exercise for pregnant women, and it has an effect on reducing maternal stress and encouraging infant attachment
Effects of music therapy on parturient anxiety; Lima et al. (2014) 1	To evaluate the effectiveness of music therapy in reducing anxiety during the first clinical period of childbirth using the methodology of clinical, controlled and randomized teaching	Brazil; 29 pregnant women; study group = 15; control group = 14	Care was not mentioned. duration: not mentioned. description: not mentioned.	State-Trait Anxiety Inventory	All pregnant women in the study group reported that undergoing music therapy was easy, and that they would use music therapy in the next labor. After the intervention, the researchers observed a decrese in the grade of anxiety from high to medium in 2 patients, and from high to low in 1 patient	When submitted to the intervention with music therapy, there was a reduction of anxiety in 3 patients in the study group. The parturients in the control group maintained the degree of anxiety throughout delivery
The effects of music listening on psychosocial stress and maternal-fetal attachment during pregnancy Chang et al. (2015) 21	To examine the effects of listening to music on psychosocial stress and maternal-fetal attachment during pregnancy	Taiwan; 2009-2010; 296 pregnant women; study group = 145; control group = 151	Individual care. Duration: 30 minutes for 2 weeks. Description: the study group received routine prenatal care and listened to music. The control group received only routine prenatal care	Pregnancy Stress Rating Scale; Perceived Stress Scale; and Maternal Fetal Attachment Scale	The results of the posttest identified a significantly lower level of psychosocial stress in the study group compared with the controls, particularly regarding the stresses related to baby care, the change in family relationships, and the identification of the maternal role	The findings support the effectiveness of listening to music in helping pregnant women cope with stress, especially pregnancy-related stress. Although this study found no effect on musical hearing on perceived general life stress or maternal-fetal attachment, the evidence indicates that music is an effective non-invasive pregnancy-related intervention for women which has minimal or no side effects and is economical and convenient
Effects of music listening on stress, anxiety, and sleep quality for sleep-disturbed pregnant women; Liu et al. (2016) 22	To examine the effects of listening to music on stress, anxiety and quality of sleep in pregnant women with sleeping disorders	Taiwan; 2014; 121 pregnant women; study group = 61; control group = 60	Individual care. Duration: 30 minutes for 2 weeks. Description: the control group received only the usual prenatal care. The study group was instructed to listen to at least 1 record (30 minutes) of the five pre-recorded CDs compiled by the researcher, or a minimum of 30 minutes of their favorite music per day at bedtime for two weeks	Pittsburgh Sleep Quality Index, Perceived Stress Scale, and State-Trait Anxiety Inventory	No statistically significant differences were identified among the 60 pregnant women with sleeping disorders in their demographic and clinical characteristics or the scores on the scales prior to the administration of musical intervention. The analysis confirmed that the posttest scores on the scales reflected significant differences from their initial scores. With all other variables controlled, the women in the study group had statistically lower scores on the scales than the controls	This study pointed out that two-week music-listening interventions can reduce stress, anxiety and improve the quality of sleep of pregnant women with sleeping disorders. The analysis of participants' diaries also suggested that mothers' choices about musical genres may be more correlated with perceived prenatal benefits or with the desire to interact with the fetus
Effect of music intervention on maternal anxiety and fetal heart rate pattern during non-stress test; Oh et al. (2016) 30	To examine the effects of musical intervention on maternal anxiety, fetal heart rate pattern, and test time during non-stress test for prenatal fetal assessment	South Korea;2013-2014; 60 pregnant women; study group = 30; control group = 30	Individual care. Duration: 20 minutes; the number of days was not mentioned. Description: the prepared songs had a time of 60 to 80 beats, and were based on the pregnant woman's heart count. The songs were divided into 5 genres, such as hymns or contemporary Christian music, classics, pop, and, with the help of music experts, a total of 25 CDs were made (5 songs of each genre). The isolated space was used to block out the noise. In the study group, after the non-stress test, the State-Trait Anxiety Inventory was applied, blood pressure, pulse and temperature were recorded, while the songs selected by the pregnant woman were played for 20 minutes. In the control group, the non-stress test was applied, but there was no music while collecting the data	State-Trait Anxiety Inventory	The study group had significantly lower scores on the anxiety scale than the controls. There were no significant differences in systolic blood pressure and pulse rate between the two groups. The baseline fetal heart rate was significantly lower in the study group than in the controls. Acceleration frequency in fetal heart rate was significantly increased in the study group compared to the controls. There were no significant differences in fetal movement and test time for reactive non-stress test between the groups	Musical intervention can be effective for anxiety during the non-stress test
Effects of prenatal music stimulation on state/trait anxiety in full-term pregnancy and its influence onchildbirth: a randomized controlled trial; García González et al. (2018) 24	To investigate the effect of music on maternal anxiety, before and after the non-stress test, and the effect of music on delivery	Spain; 2013-2014; 409 pregnant women; study group = 204; control group = 205	Individual care. Duration: 40 minutes per session; listening to music for 14 sessions, three times a week, at the same time of day. Description: the 409 pregnant women who went for routine prenatal care were randomized in the third trimester to receive music (n = 204) or no music (n = 205) stimulation during the non-stress test. The study group intervention were informed about how to listen to the music at home and received music recorded on CDs	State-Trait Anxiety Inventory	Before the non-stress test, term pregnant women who received musical intervention had a state-trait-anxiety score similar to those of the control group. After the test, the average anxiety score score of each group was recorded; study group: 30.58 ± 13.2; control group: 43.11 ( *p* < 0.001).	Prenatal music intervention can be a useful and effective tool to reduce anxiety in pregnant women at term during the non-stress test and improves the delivery process by reducing the first stage of labor
Effects of prenatal music stimulation on fetal cardiac state, newborn anthropometric measurements and vital signs of pregnant women: A randomized controlled trial; García González et al. (2017) 4	To identify the effects of prenatal musical stimulation on the vital signs of pregnant women at term, on the modification of the fetal cardiac state during the fetal monitoring cardiotocograph and on the anthropometric measurements of newborns after birth	Spain; 2013-2014; 409 pregnant women; study group = 204; control group = 205	Individual care. Duration: 40 minutes per session; listening to music for 14 sessions, 3 times a week, at the same time of day. Description: The 409 pregnant women who went for routine prenatal care were randomized in the third trimester to receive music (n = 204) or no music (n = 205) stimulation during the non-stress test. The study group were informed about how to listen to the music at home and received music recorded on CDs	Fetal cardiac status, maternal vital signs, anthropometric measurements of the fetus	The graphs showed a significant increase in FCFB and greater fetal reactivity, with accelerations of fetal heart rate in pregnant women with musical stimulation. After the fetal monitoring cardiotocograph, there was a statistically significant decrease in systolic and diastolic blood Pressure and heart rate in women in the study group	Music can be used as a tool that improves the vital signs of pregnant women during the third trimester, and can influence the fetus, increasing fetal heart rate and fetal reactivity
Effect of Turkish classical music on prenatal anxiety and satisfaction: a randomized controlled trial in pregnant women with pre-eclampsia; Toker and Kömürcü (2017) 15	To evaluate the effect of music therapy on anxiety and satisfaction in pregnant women with pre-eclampsia	Turkey; 2012-2014; 70 pregnant women; study group = 35; control group = 35	Individual care. Duration: 30 minutes, every day, for 7 days. Description: the pregnant women in the study group were subjected to a 30-minute classical Turkish music session every day for a period of 7 days (5 days before and 2 days after delivery) while the controls received routine care and were also assigned 30 minutes bedrest per day	Personal Information Form, State-Trait Anxiety Inventory, systolic and diastolic blood pressure, pulse and respiratory rate, non-stress test, fetal movements, fetal heart rate (for the first 5 days)	The differences in anxiety scores were not statistically significant ( *p* > 0.05). On the other hand, the Newcastle Satisfaction scores of the study group were higher than those of the controls ( *p* < 0.01). Finally, when considering fetal movement counts, a significant increase was determined in the study group, while music therapy reduced the fetal heart rate and blood pressure ( *p* < 0.05)	It can be suggested that nurses and midwives can use music therapy in the care and monitoring of pregnant women with pre-eclampsia in obstetric units
Prenatal singing – sound alchemy for pregnant women; Martins (2017) 19	To broaden awareness of women's body wisdom, empowerment, expression of the pregnant woman's feelings, affective communication between the pregnant woman and the baby in the womb	Brazil; 2016; 12 pregnant women	Group care. Duration: weekly, 2 hours long. Description: the methodology of the prenatal singing classes involved female songs and games of musical and vocal improvisation; sound meditations with creative visualizations; sound bath; circle singing; body breathing; and vocal exercises to prepare for childbirth; and sound improvisations with musical instruments	Not mentioned	Not mentioned	Vocal exercises were keys that opened the doors for connection with the nature of the female body. They had as objectives to release the voice and to express sensations and feelings vocally to unveil the relations among the voice, the pelvic floor and breathing, and to send affective sonic vibrations to the fetus in the womb. The experiences emphasized the affective dimension in the act of singing: the vibrational communication that the pregnant woman established in the communication with her unborn child
Prenatal listening to songs composed for pregnancy and symptoms of anxiety and depression: a pilot study; Nwebube et al. (2017) 26	To determine whether listening to specially-composed music would be an effective intervention to reduce symptoms of prenatal anxiety and depression	United Kingdom; 2014-2015; 111 pregnant women	Individual care. Duration: 20 minutes, for 12 weeks. Description: the study group listened to specially-composed songs daily, and the control group did daily relaxation. Composer Jennie Muskett wrote the songs specifically for use during pregnancy. The songs were composed using specific times, musical forms and phrases designed to induce a calm state	State-Trait Anxiety Inventory, Edinburgh Postnatal Depression Scale	The study group showed lower values of trait anxiety ( *p* = 0.0001) (effect size: 0.80), state anxiety ( *p* = 0.02) (effect size: 0.64) and Edinburgh Postnatal Depression Scale ( *p* = 0.002) at week 12 in relation to the baseline by the paired *t* -test. There were no such changes in the control group	Although this pilot study showed high levels of friction, the results suggest that listening to relaxing music regularly should be further explored as an effective non-pharmacological means to reduce anxiety and prenatal depression
State-trait anxiety levels during pregnancy and fetal parameters following intervention with music therapy; Garcia-Gonzalez et al. (2018) 7	To investigate the effect of music therapy on maternal anxiety, before and after the non-stress test, and the effect of maternal anxiety on the process of childbirth and birth	Spain; 2013-2014; 409 pregnant women; study group = 204; control group = 205	Individual care. Duration: 40 minutes per session; listening to music for 14 sessions, 3 times a week, at the same time of day. Description: the 409 pregnant women who went for routine prenatal care were randomized in the third trimester to receive music (n = 204) or no music (n = 205) stimulation during the non-stress test. The study group were informed about how to listen to the music at home and receive music recorded on CDs	STAI	After the non-stress test, the study group had significantly lower scores on state anxiety as well as trait anxiety than the controls. In addition, the study group had lower levels of trait anxiety than the controls in relation to the variables of the birth process, and greater weight at birth and breast circumference in the newborn, respectively	The intervention of music therapy during pregnancy can reduce high levels of trait anxiety during the third trimester. Further research on the influence of music therapy as an intervention on maternal anxiety and on the birth process and birth size are needed during pregnancy
**DELIVERY**						
Effect of music on labor and on the newborn; Tabarro et al. (2010) 20	To verify and describe the effects of music in the labor of women assisted in five maternities; to verify the baby's behavior and reactions, when submitted to the melodies listened to by their mothers during pregnancy and labor, through the mothers' speeches, obtained in the first three months after delivery	Brazil; 2008; initially, 87 pregnant women, but only 27 fulfilled the criteria for inclusion	Group care. Duration: from prenatal care to the postpartum period. Description: musical awareness through a portable tape player, a series of 8 to 10 melodies was made available, selected especially for the study, in an intensity compatible with the acceptance of the group. The period for this experiment ranged from 35 to 45 minutes. In each session, a different series of melodies was listened to by the same group. The groups ranged from two to nine women. The information recorded on the sheets of each pregnant woman was used to record an individualized CD that was then delivered to each future mother with the recommendation to take it to the maternity ward at the time of delivery. During the time of observation of labor, every 2 hours, the music was suppressed for a period of 30 minutes. At the end of each of these periods, the elements of control of the evolution of labor were recorded on an observation sheet	Not mentioned	Only 12 parturients had their labor accompanied by the melodies of their choice, and they were interviewed in the postpartum period. As for the effect of music on the newborns, 20 mothers were interviewed; 1 of the 12 accompanied in labor did not have her stereo during the puerperium, and could not perform the observation with her baby	Effects such as pain relief during contractions, help in reducing tension and fear, environmentalization of the parturient in the hospital, encouragement to prayer and spirituality have been reported.
Effects of music therapy on labor pain and anxiety in Taiwanese first-time mothers; Liu et al. (2010) 23	To investigate the effects of music on the reaction to pain and anxiety during labor	Taiwan; 2009; 60 pregnant women; study group = 30; control group = 30	Individual care. Duration: during delivery. Description: the study group received routine care and music therapy, while the controls received only routine care. A visual analog self-report scale for pain and a nurse assessed the behavioral intensity present to measure labor pain. Anxiety was measured with a visual analog scale for anxiety and finger temperature. Pain and anxiety between groups were compared during the latent phase (2-4 cm of cervical dilation) and active phase (5-7 cm) separately	Visual Analog Scale for Pain, Present Behavioral Intensity, Visual Analog Scale for Anxiety and Finger Temperature	In comparison with the controls, the study group presented significantly lower pain, anxiety and finger temperature during the latent phase of labor. However, no significant differences were found between the two groups in any of the outcome measures during the active phase	This study provides evidence for the use of music as an intervention for pregnant women having labor pains and anxiety during the latent phase of labor. The results confirm that listening to music is an acceptable and non-medical coping strategy for pregnant women, especially for the reduction of pain and anxiety in the initial phase of labor
Comparison between massage and music therapies to relieve the severity of labor pain; Taghinejad et al. (2010) 32	To compare the effects of massage and music therapy on the severity of labor pain	Iran; 2007; 101 pregnant women; massage group = 51; music therapy group = 50	Individual care. Duration: during the latent phase of labor. Description: pregnant women hospitalized for normal delivery were randomly divided into two groups. Pain was measured using the visual analog scale, and the two groups were compared in terms of pain intensity before and after the interventions. As soon as the cervix was dilated by up to 3-4 cm, women in the massage therapy group were asked to close their eyes and breathe rhythmically and deeply During contractions of the uterus, they were asked to breathe more deeply and more calmly, concentrating on the massage. All patients in this group received a 30-minute massage. The women in the music therapy group were asked to listen to soft traditional music (1 of 5 optional types) without lyrics, using headphones for 30 minutes, starting early in the active phase of labor	Visual Analog Scale	Mothers in the massage therapy group had a lower level of pain compared to those in the music therapy group ( *p* = 0.009). A significant difference was observed between the two groups in terms of severity of pain after the intervention ( *p* = 0.01). Labor pain was significantly relieved after therapeutic massage (p = 0.001)	Massage therapy has proven to be an effective method for reducing and relieving labor pain compared to music therapy, and can be clinically recommended as an alternative. It is a safe and affordable method of pain relief, in which the use of pharmacological or non-pharmacological methods are optional
Effect of music on labor pain relief, anxiety level and postpartum analgesic requirement: a randomized controlled clinical trial; Simavli et al. (2014) 16	To evaluate the effect of music on labor pain and anxiety, maternal hemodynamics, fetal-neonatal parameters, and the need for analgesics in the postpartum period in pregnant women	Turkey; 2012; 156 pregnant women; study group = 77; control group = 79	Individual care. Duration: during labor. Description: the study group listened to music during labor. Pain intensity and anxiety levels were measured using the Visual Analog Scale. The two groups were compared in terms of pain severity, anxiety level, maternal hemodynamics, fetal-neonatal parameters, and need for analgesics in the postpartum period	Visual Analog Scale	The study group had a lower level of pain and anxiety compared to the controls at all stages of labor. A significant difference was observed between the two groups in terms of maternal hemodynamics and fetal heart rate after the intervention. Postpartum analgesic requirement decreased significantly in the study group	Listening to music during labor has a positive impact on labor pain and anxiety, maternal-fetal parameters and the need for analgesics
Effect of music therapy during vaginal delivery on postpartum pain relief and mental health; Simavli et al. (2014) 5	To evaluate the effects of music therapy on postpartum pain, anxiety level, satisfaction, and rate of early postpartum depression	Turkey; 2012; 161 pregnant women; study group = 80; control group = 81	Individual care. Duration: during labor. Description: The study group listened to self-selected songs during labor. Postpartum pain intensity, anxiety level and satisfaction rates were measured using the Visual Analog Scale, and the postpartum depression rate was assessed using the Edinburgh Postpartum Depression Scale in postpartum days one and eight	Visual Analog Scale and Edinburgh Postnatal Depression Scale	The study group had a lower level of postpartum pain and anxiety than the controls, and this was statistically significant at all time intervals. A significant difference was observed between the two groups in terms of satisfaction rate ( *p* < 0.001) and the rate of postpartum depression on days 1 and 8	The use of music therapy during labor reduced anxiety and postpartum pain, increased satisfaction with the child's birth and reduced the rate of early postpartum depression. Music therapy can be clinically recommended as an analogous, safe, easy and pleasurable non-pharmacological method for postpartum well-being
Effects of music during multiple cesarean section delivery; Handan et al. (2018) 17	To evaluate the effects of nursing intervention using music therapy to relieve anxiety levels in pregnant women with multiple cesarean sections	Turkey; 2015-2016; 60 pregnant women; study group = 30; control group = 30	Individual care. Duration: during the c-section. Description: a list of their favorite songs was selected to be played during the c-section. They were reproduced at the desired volume of each patient throughout the surgery, using a stereo player. Physiological parameters and anxiety levels in the form of the questionnaire were recorded on the suture too. The data from the questionnaire were collected from women in the control group through interviews; their vital findings were recorded before and after anesthesia procedures, without intervention during the entire surgery	Structured questionnaire and Visual Analog Scale	The physiological indicators of anxiety and blood pressure were reduced regarding the initial values in the study group when compared to the control group	Music therapy reduces the physiological and cognitive responses of anxiety in patients undergoing multiple c-sections, and can be used in the clinical practice
Coping with preoperative anxiety in cesarean section: physiological, cognitive, and emotional effects of listening to favorite music; Kushnir et al. (2012) 31	To assess the effects of listening to music while waiting for a c-section: emotional, cognitive and stress-related physiological reactions	Israel; 2005; 60 pregnant women; study group = 28; control group = 32	Individual care. Duration: 40 minutes before c-section. Description: a list of songs of their choice was selected. The study group listened to selected songs using a headset 40 minutes before the c-section.	Mood State Scale; Perceived Threat of surgery scale; vital signs	The study group experienced a significant increase in positive emotions and a significant decline in negative emotions and perceived threat of the situation when compared to the controls, who exhibited a decline in positive emotions, an increase in perceived threat of the situation, and no change in negative emotions. The study group also exhibited a significant decrease in systolic blood pressure compared to a significant increase in sdisatolic blood pressure and RF in the controls	Listening to your favorite music just before a c-section can be an economic and emotionally-focused coping strategy
**POSTPARTUM PERIOD**						
The efficiency and duration of the analgesic effects of musical therapy on postoperative pain; Sen et al. (2010) 18	First, to discover the effect of music therapy on postoperative analgesia, and, secondly, to determine the duration of its effect.	Turkey; 2009; 70 pregnant women; group 1 with music = 35; group 2 with no music = 35	Individual care. Duration: 1 hour after surgery. Description: pregnant women who underwent c-sections were included and randomly allocated to two groups as follows: in group 1, pregnant women listened to music through a headset for an hour after surgery, while in group 2, they did not listen to any music during the same period. In the postanesthetic care unit, pregnant women were connected to a patient-controlled analgesia device (tramadol 3 mg/ml), which was adjusted to deliver a 20 mg bolus, with a 15 min blocking interval and a maximum 4-hour dose of 150mg. Postoperative pain was assessed using the visual analog scale and tramadol consumption was recorded at 4, 8, 12, 16, 20 and 24 hours	Visual Analog Scale	There was a significant decrease in group 1 in relation to the frequency of analgesic delivery in the 4th postoperative hour. Regarding the consumption of tramadol in the postoperative period, the values measured in the fourth hour were significantly lower in group 1. The total amount of tramadol consumption and additional analgesic use in the 24-hour postoperative period were again lower in group 1 when compared to group 2. All scores on the Visual Analog Scale were lower in group 1 when compared to those of group 2	Music therapy provided after surgery reduces postoperative pain in the first 24 hours and analgesic consumption in the first 4 hours

The articles regarding the prenatal period, delivery, and the postpartum period report relaxation, decreased levels of anxiety, psychosocial stress, depression and pain, increased maternal bond with the baby, improved quality of sleep, control of fetal heart rate and maternal blood pressure, and decreased drug intake in the postoperative period. According to Carvalho, 33 music stimulates action and emotional expression in individuals, and prompts them to control states of physical and psychological homeostasis, having effects on physiology, behavior, cognition, emotions, and social interaction. 33

Regarding the prenatal period, 15 articles were analyzed, and 9 of them were relevant for the present review, for they dealt with anxiety in parturient women, and 4 out of these 9 studies were carried out during the nonstress test. According to Primo and Amorim, 34 during pregnancy women may experience anguish and anxiety due to the need to adapt to situations regarding maternity.

As for childbirth, seven articles were analyzed; four of them were related to pain during labor, six dealt with anxiety, and two reported a significant reduction in blood pressure. In the study by Gayeski and Brüggemann, 35 the perception of mothers regarding non-pharmacological methods for pain relief, the feeling of well-being, an having emotional support were reported to facilitate the parturition process. The authors state that there is a need to expand information on these methods throughout pregnancy, and they point out that there are more investigative studies on the use of these non-pharmacological methods for pain relief in women in labor, which aim to improve humanized actions in assisting parturient women, resstablishing the autonomy of women regarding labor and birth. 35

As for the results found, we could not perform an in-depth analysis of the methodology, since some studies were inaccurate, omitted data, and/or presented vague information. In addition to the incomplete methodology, some of the studies selected do not inform if they were conducted by music therapists, and most of them were performed by other health professionals. As a result, these studies did not have a theoretical framework for music therapy and did not follow a validated protocol. Low methodological quality was a common finding among systematic reviews that examine music-based interventions, with variations between the number of interventions and the duration of each session, which can interfere with the results, limiting the benefits that the pregnant woman and her fetus could obtain; therefore, it is necessary to think about comprehensive interventions that cover the prenatal, delivery and postpartum periods. 8

Carvalho 33 states that, in the practice of music therapy, music is not therapeutic, and is not used as an end in itself, but becomes a mediator of therapeutic individual or group relationship guided by a qualified and certified music therapist. It is important to remember that music therapy is included among the services provided by the Brazilian Unified Health System. In addition to maintaining its autonomy, it develops a practice consistent with the principles, seeking the necessary transformations, and without restricting its vision. The conviction regarding the contribution of studies on music therapy for the medical field brings another level of scientific knowledge necessary for the development of music therapy, thus answering existing questions. The need for the area to discuss some concepts is understood, contributing to clinical practice in different contexts. 36

Another aspect to be observed was that most of the studies found were conducted outside Brazil (only three Brazilian publications were found), which suggests that further studies in this area should be carried out. Based on the results of previous studies, Brandalise 37 states that there are few music therapists who publish articles and books reporting their findings and professional experiences, suggesting that there should be an incentive and preparation for the professional to engage in research.

## Conclusion

From the results obtained, we can concluded that the performance of music therapy during the prenatal, delivery and postpartum periods can provide several benefits to the pregnant woman and the fetus, thus justifying its importance in this field. There is a demand in the job market for more professional music therapists, as well as for more studies on this subject performed by these professionals.
